# Consistency of Continuous Ambulatory Interstitial Glucose Monitoring Sensors

**DOI:** 10.3390/bios8020049

**Published:** 2018-05-16

**Authors:** Pei T. Wu, David E. Segovia, Cathy C. Lee, Kim-Lien Nguyen

**Affiliations:** 1 Division of Cardiology, David Geffen School of Medicine at UCLA and VA Greater Los Angeles Healthcare System, Los Angeles, CA 90024, USA; ptwu@ucla.edu; 2 Department of Geriatrics, David Geffen School of Medicine at UCLA and Geriatric Research Education and Clinical Center (GRECC), VA Greater Los Angeles Healthcare System, Los Angeles, CA 90095, USA; David.Segovia@va.gov (D.E.S.); cathy.c.lee@ucla.edu (C.C.L.)

**Keywords:** continuous glucose monitoring, interstitial glucose sensors, intra-subject agreement, postprandial glucose

## Abstract

Aims: The abdominal region is the most common location for continuous glucose monitor (CGM) sensor insertion. However, a paucity of post-marketing data is available to demonstrate intra-individual consistency of CGM readings at different abdominal insertion sites. Methods: Healthy adults (fasting glucose (FG) < 5.5 mmol/L; BMI < 30 kg/m^2^) were recruited and a CGM sensor was placed on each side of the abdomen. Postprandial and continuous 48-h interstitial glucose levels were analyzed. Results: There was no significant difference in the 3-h postprandial glucose (PPG) level derived from the left versus right CGM, which remained non-significant after adjusting for waist circumference or FG. Among the glucose levels recorded over 48-h, values on the left site were greater in 3.6% of the data points (*p* < 0.05). After adjusting for waist circumference, only 0.5% of the glucose values remained significantly greater on the left (*p* < 0.05). When adjusted for FG, similar results were observed. For both PPG and 48-h readings, the mean absolute relative difference was not significant between the two abdominal sites. Conclusions: CGM-derived glucose measures were highly consistent between the left and right abdomen during both the postprandial and post-absorptive periods.

## 1. Introduction

Continuous glucose monitoring (CGM) systems have changed the practice of outpatient glucose monitoring by facilitating more patient-centered or patient-specific care through real-time measurement of interstitial glucose levels. In clinical practice, CGM devices are frequently used in patients with labile diabetes in order to define patterns of interstitial glucose concentration, which changes continuously [1]. More importantly, however, CGMs have enabled clinicians and researchers to assess the full spectrum of glycemic variation under everyday life conditions, including meal times, activity, and during sleep. Compared to conventional glucose meters, which intermittently measure capillary blood glucose concentration, CGMs provide information about changes in interstitial glucose levels throughout the day. Results from ambulatory CGMs help clinicians obtain comprehensive glycemic patterns. Information, such as direction, magnitude and rate of change in glucose levels, can be mapped and used to help patients improve their glycemic control, particularly those with brittle diabetes [2,3].

Prior work showed that the accuracy of CGMs was not affected by sensor insertion sites, such as abdomen versus triceps [4] and abdomen versus upper arm [5,6]. While King et al. [5] have reported high agreement between left and right abdominal CGM readings in individuals with type 1 diabetes, the analyses were based on the rate of glucose change from one set of abdominal CGM readings over a 24-h period. Variations in glycemic pattern and their relationship to daily events were not evaluated. For example, whether postprandial hyperglycemia (a daily event with large glucose fluctuations) is related to lower agreement between CGMs is unclear. Furthermore, rotating sensor insertion sites were suggested to minimize tissue irritation and prevent infection [7] and insulin-induced lipohypertrophy [8]; however, the degree of agreement between different abdominal insertion sites remains unknown.

The purpose of the present study was to evaluate the consistency of CGM readings at different abdominal insertion sites in adults with normal fasting glucose levels by comparing CGM postprandial readings and over a 48-h period.

## 2. Methods

All study protocols and consent forms were in agreement with the Declaration of Helsinki and were approved by the Institutional Review Board. Study protocols were in compliance with the Health Insurance Portability and Accountability Act. All subjects provided written informed consent.

### 2.1. Study Participants

Healthy female and male volunteers (ages 18–55 years) were screened. Inclusion criteria were: (1) fasting glucose level < 5.5 mmol/L and (2) body mass index (BMI) < 30 kg/m^2^. Fasting glucose level was measured after 10-h of overnight fasting. Self-reported dominant hand and leg, as well as sleep position (right, left, back, abdomen), were recorded. Potential participants were excluded if they (1) were pregnant; (2) were smokers; (3) had hypertension, defined as systolic blood pressure ≥ 140 mmHg or diastolic blood pressure ≥ 80 mmHg; (4) had used medications that influence metabolic function (e.g., insulin, metformin) in the prior 6 months; or (5) had diabetes. A total of 10 volunteers enrolled in the study.

### 2.2. Quantitative Measurements

#### 2.2.1. Anthropometric Measurements

Height, weight, and waist circumference (WC) were measured in duplicate and averaged. BMI was defined as body mass (kg) divided by the square of the body height (m^2^).

#### 2.2.2. Capillary Blood Glucose and Continuous Interstitial Glucose Measurement

The CGM device (iPro^®^2, Medtronic MiniMed, Northridge, CA, USA) consists of a transmitter and a sensor. The sensor (Sof-Sensor^®^, Medtronic MiniMed, Northridge, CA, USA) contains glucose oxidase and oxidizes glucose to gluconolactone while reducing oxygen to hydrogen peroxide. When peroxide reacts with platinum inside the sensor, an electrical signal is generated and sent by wireless radiofrequency telemetry to the transmitter. The electrical signal is then converted into a glucose reading by a computer program.

To simultaneously measure continuous, 48-h interstitial glucose levels, all participants wore two CGMs concurrently; each CGM was connected to their respective glucose sensors placed on each side of the abdomen. The glucose sensor was inserted into the subcutaneous abdominal adipose tissue (approximately 7 and 10 cm to the left and right lateral aspect of the umbilicus). Interstitial glucose concentration was recorded by the sensors at 5-min intervals. An adhesive waterproof dressing film was then applied on the insertion area to cover both the glucose sensor and recorder. A single member of the research team inserted and positioned the device. This procedure has been previously validated [9].

All participants received detailed instructions on wearing the CGM devices. Each subject performed CGM calibrations by taking at least five separate capillary blood glucose meter readings per day (immediately after waking up and before breakfast, lunch, dinner, and bedtime). To avoid interference with the 3-h postprandial glucose analysis, subjects were instructed to have meals at least 3 h apart. Meals were consumed at the following designated time periods: breakfast 7–8 a.m., lunch 12–1 p.m., and dinner 6–7 p.m. A log sheet was provided to each participant to record meals, meal time, capillary blood glucose level, and exercise or other strenuous physical activity (defined as activities greater than 6 metabolic equivalents, such as hiking or carrying a heavy load). After the 48-h monitoring period, CGMs were removed and de-identified data from the recorder were uploaded to CareLink iPro via a secured internet connection.

Capillary blood glucose measurements derived from conventional glucose meters served as the reference standard. A glucose meter (ACCU-CHEK Aviva Plus System, Roche Diabetes Care, Inc. Indianapolis, IN, USA), test strips, and one-time-use lancets were provided to participants. CGM sensor calibration was performed as described on the manufacturer’s website [10] and by Gross et al. [11]. To calibrate CGMs, each subject was asked to perform finger sticks five times per day.

### 2.3. Assessment of Glycemic Variability

Interstitial glucose readings from CGMs at the left versus right abdominal sites were compared and analyzed in two parts. The capillary blood glucose level measured by the glucose meter at 0-min of each meal was paired with the 0-min postprandial glucose (PPG) level from the CGM recorder.

#### 2.3.1. Postprandial Glucose (PPG) Excursion

Based on the participants’ CGM meal time log sheets, a total of six meals over the course of 48 h was identified. PPG values at 30-min intervals (0, 30, 60, 90, 120, 150, and 180 min after each meal) and the peak PPG at 2 h and 3 h post meal time (Peak PPG_2h_ and Peak PPG_3h_, respectively) were collected. The change in PPG (∆PPG) at 2 and 3 h after each meal was calculated (∆PPG_2h_ and ∆PPG_3h_, respectively). The coefficient of variation (CV) at 2 and 3 h after each meal was calculated (CV_2h_ and CV_3h_, respectively) as an indicator of the sensor consistency and precision. Using the trapezoidal rule, the area under the PPG curve (AUC) at 2 h and 3 h post meal time (PPG AUC_2h_ and PPG AUC_3h_, respectively), which reflected glycemic response to foods, was derived. The mean absolute relative difference (MARD) reflected the difference between CGM-derived and glucose-meter-derived glucose levels and was calculated as:

*MARD* (%) = [|*CGM_glucose_* − *Glucose meter_glucose_*|/(*Glucose meter_glucose_*)] × 100%. (1)

For each variable, values among completed participants were averaged and the mean was used for statistical analyses.

#### 2.3.2. Dynamic, Continuous 48-h Interstitial Glucose Profile

A total of 576 paired data points (288 data points per 24-h period, left and right abdominal sites) was assessed. Mean, minimal, and maximal values of glucose level; counts and duration of excursion for glucose level >7.8 mmol/L or <4.0 mmol/L; coefficient of variation (CV); and the area under the glucose curve (AUC 48 h) were collected. Five glucometer-derived glucose readings for each day were paired with CGM readings. The difference between CGM-derived and glucometer-derived glucose levels was calculated and MARD was computed according to Equation (1) (above). The differences between left and right CGMs were calculated by subtracting the value of the right CGM reading from the left CGM reading (CGM_left_−CGM_right_) for each paired data point. The differences were then averaged for each of the 576 paired data points.

### 2.4. Statistical Analyses

Statistical analyses were performed using SPSS version 20.0 (IBM Corp., Armonk, NY, USA) and MATLAB R2016b version 9.1.0.441655 (The MathWorks, Inc., Natick, MA, USA). Normality of data distribution was determined using the Shapiro–Wilk test. Descriptive statistics are reported as mean ± standard error of the mean (SEM). Comparisons and agreements between abdominal sites were performed using the Wilcoxon signed-rank test and Bland–Altman analysis. The Clarke Error Grid analysis [12] was used to test for consistency/discrepancy between readings from the two abdominal sites. The Quade’s rank analysis of covariance was performed to adjust for waist circumference or fasting glucose levels. A two-tailed alpha value of 0.05 was considered significant.

## 3. Results

Among the 10 recruited volunteers, one subject declined further participation due to skin irritation to the Tegaderm film. A total of nine healthy volunteers (32.6 ± 4.1 years; four men) were included in the analyses. Participant characteristics are presented in Table 1. Overall, there were no glucose levels <4.0 mmol/L. For glucose levels >4.0 mmol/L, 97.4% of the left CGM readings and 97.4% of the right CGM readings were within ±20% of readings from a conventional glucose meter.

Among the nine subjects who completed the study, eight subjects provided responses on their dominant hand and leg and preferred sleep position.

### 3.1. Postprandial Glucose Level

**Figure 1:**
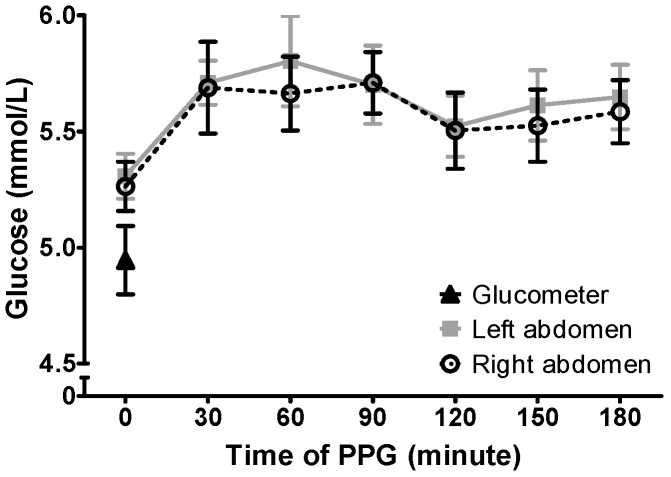
Postprandial Glucose (PPG) Levels by Conventional Glucose Meter and Continuous Glucose Monitoring Systems (CGMs). Blood glucose levels were measured at 0 min of each meal using a conventional glucose meter. The interstitial glucose level was collected at 0, 30, 60, 90, 120, 150, and 180 min after each meal using the CGMs on left and right abdominal sites. No significant difference was found between the left versus right CGM (*p* > 0.05).

Postprandial glucose levels at 30-min intervals between 0 and 180 min are presented in Figure 1. No significant difference in glucose levels between the left versus the right side of the abdomen was observed (all *p* > 0.05).

There was no statistical difference in the ∆PPG, peak PPG, CV, and AUC (all *p* > 0.05, Table 2). Differences between CGM readings, glucose meter readings, and MARD values at the two abdominal sites were also not significant (all *p* > 0.05) (Table 2). Postprandial MARD values at both abdominal sites were within the 5% precision criteria of accuracy recommended by the American Diabetes Association [13].

Variations in glucose readings at the two abdominal sites remained non-significant after adjusting for waist circumference or fasting glucose level. These findings suggest consistent postprandial glucose level measurements at both the left and right abdominal sites, which were not influenced by the individual’s waist circumference or fasting glucose level.

### 3.2. Continuous 48-h Glucose Level

**Figure 2:**
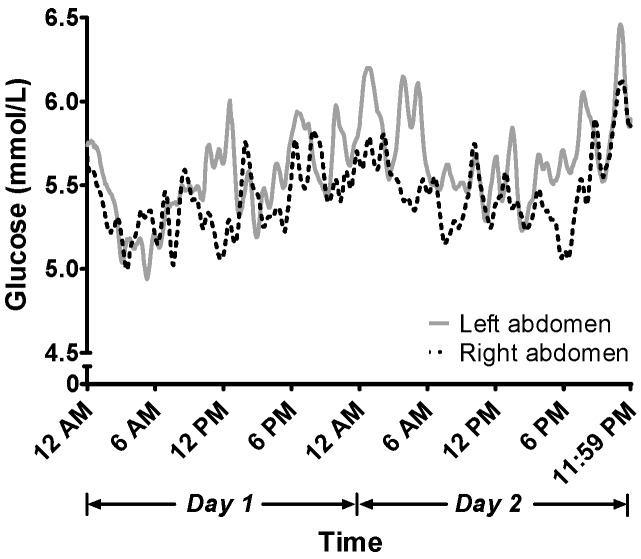
Continuous 48-h Glucose Levels by CGM. The interstitial glucose level was collected at 5-min intervals by the CGMs for a 48-h period (12:00 a.m. to 11:59 p.m. for 2 consecutive days). The grey solid line indicates the CGM on the left abdomen, and the black dotted line indicates the CGM on the right abdomen.

Continuous glucose levels over a 48-h period (00:00 a.m.–11:59 p.m. each day for 2 days) are shown in Figure 2. Among the 576 data points, significant differences in glucose levels between the two abdominal sites were observed in 23 data points (all *p* < 0.05) or approximately 4% of the collected data points. Notably, in 21 of the 23 data points, continuous glucose levels derived from right-sided CGMs were significantly lower than those measured by left-sided CGMs.

**Figure 3:**
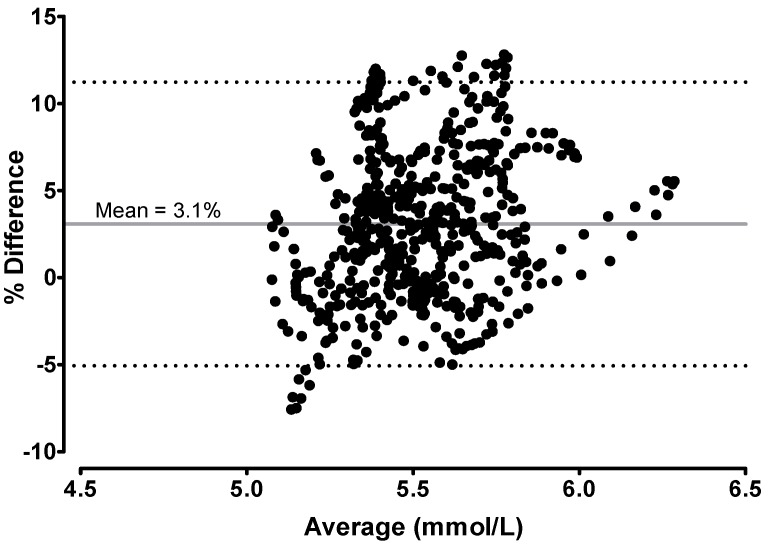
Agreement between the Left and Right CGMs. The mean (*x*-axis) and % difference (*y*-axis) of the glucose levels between the left and right CGMs were paired and plotted over a 48-h period. The grey solid line indicates the average (or bias, 3.08%) of the % differences between two CGMs; the black dotted lines indicate the 95% limits of agreement (upper limit = 11.22%; lower limit = −5.07%) between the two CGMs. The standard deviation of the % difference was 4.16.

**Figure 4:**
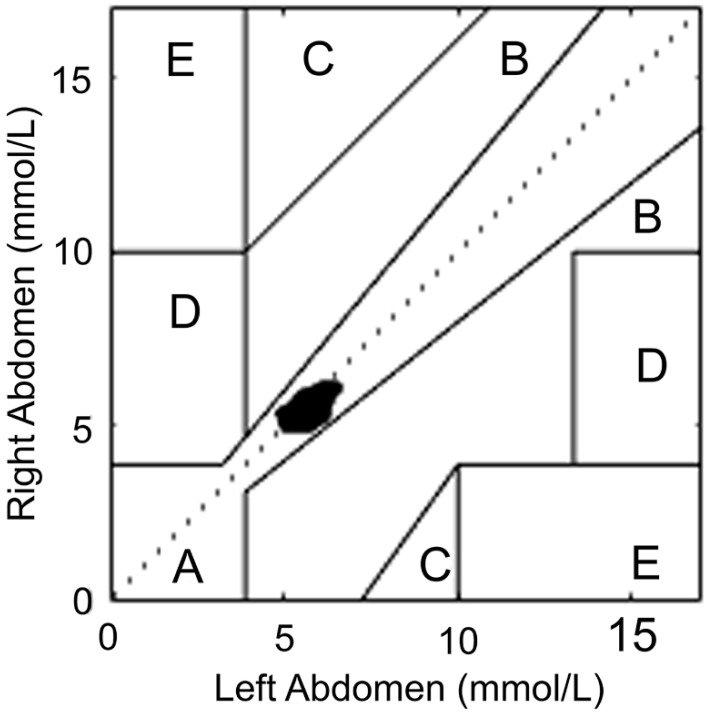
Clarke Error Grid Analysis between the Left and Right CGMs. When left and right CGM readings were paired, all data points (100%) fell into Clarke Zone A, indicating that those values were within 20% of the discrepancy. Zone B: >20% discrepancy but would not lead to inappropriate treatment; Zone C: values leading to unnecessary treatment; Zone D: potential dangerous failures to detect hypoglycemia or hyperglycemia; Zone E: confusing treatment of hypoglycemia for hyperglycemia and vice versa.

The difference between right and left CGMs is presented in Figure 3. The average of the differences (bias) between left and right CGMs was 0.17 mmol/L, indicating that on average the left CGM measures 3.08% greater than the right CGM. However, when the glucose readings were paired between the left and right CGMs, all 576 data points fell into Clarke Zone A, suggesting a <20% discrepancy between the two CGMs (Figure 4).

There were no significant differences in the average, minimal, and maximal glucose levels, number and duration of excursion >7.8 mmol/L or <4.0 mmol/L, CV, AUC, differences between glucose levels measured using CGMs and conventional glucose meters, or MARD (Table 3). Continuous 48-h interstitial MARD values at both abdominal sites were within the 10% precision criteria of accuracy recommended by the American Diabetes Association [13].

After adjusting for waist circumference, 3 of the 576 data points (0.5%) had lower CGM-derived glucose levels on the right compared to the left abdominal site (all *p* < 0.05). Similar findings were observed in 4 of the 576 data points (0.6%) after adjusting for fasting glucose level (all *p* < 0.05). These results indicate that an individual’s waist circumference and/or fasting glucose level could be potential contributors to the measured differences in glucose levels between the left and right abdominal sites.

## 4. Discussion

The present study compared the consistency of CGM-derived postprandial and 48-h continuous glucose measurements between two abdominal sites. The findings demonstrated that (1) the measurement of postprandial glucose level using CGMs is consistent between abdominal sites; and (2) the right abdominal site (compared to the left abdominal site at the level of the umbilicus) tended to have a lower glucose level when CGMs were used for 48 h. Differences in intra-individual interstitial glucose levels assessed by CGMs may be affected by waist circumference and fasting glucose levels. Lastly, postprandial and continuous 48-h MARD levels at both abdominal sites were within the 10% precision criteria for accuracy as recommended by the American Diabetic Association [13] and well below the 15% recommended by the International Organization for Standardization [14,15]; furthermore, the 48-h MARD values were comparable to the accuracy of the iPro2 CGM system, which was reported by the U.S. Food and Drug Administration as being approximately 10.1% [16].

In the present study, there were no significant differences in postprandial glucose level between the two CGMs. Consistent CGM-derived glucose measurements were observed in 96% of the collected data points over a 48-h period. These results were consistent with prior findings [4,5] and can be explained by the minimal rate of change in postprandial glucose level (±0.06 mmol/L/min) [6] and the increased trend duration [5].

Although interstitial glucose levels from CGMs both showed <10% deviation from the glucometer, notably, 4% of the collected data points showed discrepancies between CGM-derived glucose levels on the left versus right abdominal regions. Measurements from the right abdominal site tended to be lower than those from the left abdominal site. The intra-sensor precision (or CV) was comparable between left- and right-side CGMs, which both agreed with a prior study [17], indicating that the observed differences between abdominal sides were less likely due to artifacts of the device. King et al. [5] reported a similar observation, where glucose values <3.9 mmol/L were more common on the right abdomen. Further, when glucose values were <3.9 mmol/L on the left abdomen, the duration of sustained hypoglycemia was shorter. Studies involving sleep [18,19,20] and belt compression [21] on sensors have suggested a possible relationship between the performance of sensors and the pressure exerted on the sensors, specifically the potential for local decreases in blood flow due to compression. Similarly, Mensh et al. [22] demonstrated that the susceptibility of CGMs to aberrant nocturnal glucose readings was related to sleeping positions. In our study, however, dominant hand/leg or sleep position did not seem to fully explain the findings.

Local physiologic factors, such as differential capillary networks at local abdominal areas [23], variation in adipose tissue distribution [23], and regional adipose tissue blood flow [24] (along with associated local insulin response), may have contributed to the observed discrepancy in left versus right abdominal CGM readings. Others have posited subcutaneous adipose tissue thickness as a possible contributor to greater accuracy of CGM readings in subjects with higher BMI compared to those with lower BMI [6]. Waist circumference was used in the current study as a surrogate marker of subcutaneous adipose thickness; however, the findings only partially explained the discrepancy between measurements at the two sites. Due to the small number of subjects in the study, we did not observe a strong association between the waist circumference and the consistency of the CGMs (data not shown). Subcutaneous adipose tissue blood flow has been shown to vary in different abdominal regions [24], which may result in differential subcutaneous absorption of insulin and discrepancies between CGM readings. Future work to correlate subcutaneous adipose tissue distribution and/or regional adipose tissue blood flow with the accuracy of CGMs in large multi-center studies would be helpful.

The biocompatibility of the sensor itself can contribute to the discrepancy between sites. Sensor insertion causes different levels of trauma to the insertion site, which provokes inflammatory reactions. Inflammatory cells can consume glucose around the implanted sensor [25]; during wound healing, neoangiogenesis in the form of capillaries may supply additional glucose at the insertion site [25]. Sensors can also be damaged by local proteolytic enzymes and free radicals [26]. Overall, mechanisms to explain these discrepancies remain unclear and further research is also needed to better understand interstitial fluid physiology in relationship to sensor responses, particularly vascular properties, local cellular metabolism and/or lymphatics, and local wound responses.

Other factors which may affect the variation in glucose levels include food consumption and physical activity. While food consumption frequently equates to greater rates of glucose level change, the 2- and 3-h postprandial glucose levels were comparable between the two CGM sites. Unreported physical activity may also affect glucose level fluctuation. However, both food consumption and physical activity would tend to cause systemic changes in glucose levels and are therefore less likely to contribute to the observed discrepancy between sensor sites.

Given the magnitude of the approximately 3% difference in CGM readings between the two abdominal sites and all of the data points falling into Clarke Zone A, we do not suggest wearing two sensors for the purpose of glucose monitoring. This is also supported by the findings by King et al. [5], where CGM readings were compared between the arm versus abdomen in subjects with type 1 diabetes (insulin-pump treated); they reported acceptable agreement between CGM readings from different sites. However, it should be noted that in those with ectopic lipodystrophy due to other comorbid cardiometabolic conditions, our findings may not apply. Until further large-scale comparative studies are conducted in type 1 diabetic patients, it may be helpful for patients to report the site where the meter was inserted.

With a small sample size, larger clinical trials nested within the context of clinical care as pragmatic clinical trials are warranted to confirm the findings. All participants had a normal fasting glucose level (<5.5 mmol/L). Adjusting for fasting glucose levels reduced the number of discrepant CGM-derived glucose levels. This finding suggests that CGM consistency may vary among patients with different degrees of insulin sensitivity (i.e., brittle diabetes versus diabetes versus pre-diabetes versus non-diabetics). Although standard meals were not provided, participants were instructed to avoid sudden changes in their regular dietary pattern and to ensure that measurements reflected a “real-life” setting as much as possible. The participants were also instructed to consume meals within certain periods of time during the day and to report the exact time points for CGM calibrations in order to minimize inter-participant variation. Because this study was designed to compare the consistency of CGM readings at two abdominal regions rather than to assess the accuracy of the CGM system, an external glucose reference (e.g., plasma glucose level) was not included. Since glucose sensors measure glucose levels by oxidizing glucose to hydrogen peroxide, it is inevitable that some mediators, such as ascorbic acid, uric acid, and acetaminophen and salicylic acid, may contribute to deviations in the measurements by nonspecifically oxidizing hydrogen peroxide [27].

## 5. Conclusions

Continuous glucose monitoring systems allow clinicians to collect interstitial glucose levels throughout the day and night and provide continuous data points compared to conventional methods of glucose measurement. For research purposes, CGMs may be used to assess ambulatory glucose dynamics in a variety of interventions, activities, and situations. Our results indicate high agreement of CGM readings between the left and right abdominal sites. However, the readings may be partially affected by waist circumference and fasting glucose level. Higher glucose readings on the left abdomen, compared to the right abdomen, were noted and should be confirmed in larger clinical trials. As the field of big data becomes more established, deep learning algorithms may be used in concert with reported CGM results to facilitate a more individualized approach to managing postprandial glucose responses and to provide individualized trends of glycemic variability throughout the course of a patient’s disease spectrum.

## Figures and Tables

**Table 1:** Participant Characteristics.

Participant Characteristics (N = 9)	
Age (years)	32.6 ± 4.1
Gender (N)	4 men
Weight (kg)	71.5 ± 3.5
Height (cm)	168.7 ± 2.9
BMI (kg/m^2^)	25.0 ± 0.6
Waist Circumference (cm)	81.7 ± 3.8
Fasting Glucose (mmol/L)	5.0 ± 0.2
Dominant Hand (N)	
Right	8
Left	0
Dominant Leg (N)	
Right	8
Left	0
Sleep Position (N)	
Right side	4
Left side	2
Back	1
Stomach	1

BMI, body mass index.

**Table 2:** Postprandial Glucose Levels Obtained at Left versus Right Abdominal Sites Using CGMs.

Variables	Left Site	Right Site
Peak PPG_2h_ (mmol/L)	6.26 ± 0.19	6.27 ± 0.18
Peak PPG_3h_ (mmol/L)	6.38 ± 0.21	6.39 ± 0.21
∆PPG_2h_ (mmol/L)	0.34 ± 0.11	0.34 ± 0.08
∆PPG_3h_ (mmol/L)	0.33 ± 0.11	0.32 ± 0.08
CV_2h_ (%)	7.03 ± 1.12	7.37 ± 1.06
CV_3h_ (%)	7.47 ± 1.16	8.26 ± 1.12
PPG AUC_2h_ (min·mmol/L)	707.33 ± 15.30	701.53 ± 15.79
PPGAUC_3h_ (min·mmol/L)	1015.03 ± 22.91	1005.96 ± 22.68
CGM-Glucometer (mmol/L)	0.08 ± 0.04	0.03 ± 0.07
MARD (%)	2.0 ± 0.5	2.9 ± 1.1

Peak PPG_2h_, peak postprandial glucose level within 2 h after each meal; Peak PPG_3h_, peak postprandial glucose level within 3 h after each meal; ∆PPG_2h_, the averaged change of postprandial glucose level within 2 h after each meal; ∆PPG_3h_, the averaged change of postprandial glucose level within 3 h after each meal; CV_2h_, the coefficient of variation within 2 h after each meal; CV_3h_, the coefficient of variation within 3 h after each meal; PPG AUC_2h_, the area under the postprandial glucose curve for 2 h after each meal; PPG AUC_3h_, the area under the postprandial glucose curve for 3 h after each meal; CGM-glucose meter, the difference of glucose level between the CGM recorder and glucose meter at 0 min of each meal; MARD; mean absolute relative difference. All *p* > 0.05.

**Table 3:** Continuous 48-h Glucose Levels Obtained at Left versus Right Abdominal Sites Using CGMs.

Variables	Left Site	Right Site
Average Glucose (mmol/L)	5.58 ± 0.29	5.48 ± 0.08
Min Glucose (mmol/L)	4.14 ± 0.17	3.84 ± 0.18
Max Glucose (mmol/L)	7.20 ± 0.29	7.00 ± 0.24
Number of Excursion above 140 mmol/L	0.04 ± 0.04	0.02 ± 0.02
Number of Excursion below 72 mmol/L	0.03 ± 0.01	0.04 ± 0.01
Duration of Excursion above 140 mmol/L(min)	1.63 ± 1.47	0.62 ± 0.62
Duration of Excursion below 72 mmol/L(min)	1.02 ± 0.55	1.98 ± 0.91
CV (%)	10.34 ± 1.34	10.98 ± 0.71
AUC_48h_ (min·mmol/L)	15,974.05 ± 251.73	15,740.91 ± 241.12
CGM-Glucose meter (mmol/L)	−0.006 ± 0.02	−0.008 ± 0.03
MARD (%)	6.9 ± 1.2	8.1 ± 0.8

Min Glucose, the lowest value of glucose level during the 48-h monitoring; Max Glucose, the highest value of glucose level during the 48-h monitoring; CV, coefficient of variation during the 48-h monitoring; AUC_48h_, the area under the glucose curve during the 48 h; CGM-glucose meter, the difference of glucose level between the CGM recorder and glucose meter; MARD; mean absolute relative difference. All *p* > 0.05.

## Abbreviations

AUC, area under the curve; BMI, body mass index; CGM, continuous glucose monitors; MARD, mean absolute relative difference; PPG, postprandial glucose; WC, waist circumference; ∆PPG, the change in postprandial glucose.
